# Feasibility of flattening filter free beams for hippocampal avoidance whole-brain radiotherapy: a dosimetric and radiobiological analysis

**DOI:** 10.3389/fonc.2023.1290434

**Published:** 2023-11-22

**Authors:** Fangyu Liu, Yu Peng, Qian Li, Qianru Zhang, Hongyun Shi, Shuai Qie, Ruohui Zhang

**Affiliations:** ^1^ Department of Radiotherapy, Affiliated Hospital of Hebei University, Baoding, Hebei, China; ^2^ School of Nuclear Science and Technology, School of Energy and Power Engineering, Xi'an Jiaotong University, Xi'an, Shaanxi, China; ^3^ Department of Pediatrics, Affiliated Hospital of Hebei University, Baoding, Hebei, China; ^4^ Department of Radiotherapy, The Fourth Hospital of Hebei Medical University, Shijiazhuang, Hebei, China

**Keywords:** flattening filter free, hippocampal avoidance, whole-brain radiation therapy, volumetric modulated arc therapy, intensity modulated radiation therapy, normal tissue complication probability

## Abstract

**Objectives:**

The purpose of this study is to evaluate the potential of the flattening filter free (FFF) mode of a linear accelerator for patients with hippocampal avoidance whole-brain radiotherapy (HA-WBRT) by comparison with flattened beams (FF) technique in the application of volumetric modulated arc therapy (VMAT) and intensity modulated radiation therapy (IMRT) using dosimetric and radiobiological indexes based on the volume of hippocampus and target.

**Methods:**

2 VMAT- and 2 IMRT- plans were optimized in Eclipse planning system with 2 different delivery modes (6 MV standard vs. 6 MV FFF) for each of 25 patients. Dose distributions of the target and organs at risk (OARs), normal tissue complication probability (NTCP) of the hippocampus, monitor units, treatment time and quality assurance results were evaluated to compare the normal and FFF beam characteristics by Wilcoxon matched-pair signed-rank test with a significance level of 0.05.

**Results:**

VMAT-FFF provided the significantly best homogeneity and conformity of the target, delivered the lowest dose to hippocampus and the other OARs, and led to the lowest NTCP of the hippocampus among all modalities, which has the potential to alleviate neurocognitive decline after WBRT. IMRT-FFF reduced the dose to the lens with similar dose distributions of the target compared with IMRT-FF, whereas the lower dose to the hippocampus was achieved using the conventional beams. The monitor units were obviously increased by 19.2% for VMAT and 33.8% for IMRT, when FFF beams w ere used. The removal of flattening filter for IMRT resulted in a 26% reduction in treatment time, but VMAT had the similar treatment time for the two modes owing to the limitation of gantry rotation speed. Gamma analysis showed an excellent agreement for all plans at 3%/2 mm, and no statistical differences were found between FF and FFF.

**Conclusion:**

In conclusion, this study suggests that FFF mode is feasible and advantageous in HA-WBRT and VMAT-FFF is the optimal solution in terms of dose distribution of the target, OARs sparing, NTCP of the hippocampus and delivery efficiency compared to the other three techniques. Additionally, the advantages of the FFF technique for VMAT are more prominent in cases with small hippocampal volumes.

## 1 Introduction

Brain metastases represent an important clinical problem, accounting for approximately 25–45% of cancer patients, which cause significant morbidity and mortality, and management focuses on improving survival and optimizing quality of life. Whole-brain radiation therapy (WBRT) is usually the primary treatment option for patients with brain metastases, controlling macroscopic and microscopic tumor deposits within the affected area. However, it is reported that WBRT can cause long-term serious and irreversible toxic effects, including neurocognitive deterioration, leukoencephalopathy, cerebellar dysfunction and dementia, potentially compromising patients’ quality of life (1–3). In the last few decades, it has already been proven that hippocampus is crucial to memory function (4). Furthermore, recent studies have indicated that the neurocognitive decline (short and long-term memory loss and cognitive impairment) is strongly associated with radiation-induced injury to the neural stem cells in the subgranular zone of the hippocampi (5, 6).

With innovative techniques for planning and delivering WBRT, such as volumetric modulated arc therapy (VMAT) and helical tomotherapy, allowing a better sparing of organs at risk (OARs), it is possible to selectively spare sensitive brain regions, such as the hippocampus, while maintaining uniform dose delivery to the remaining brain. Hence, hippocampal avoidance (HA) during whole brain radiotherapy (HA-WBRT) has become an emerging strategy that is expected to mitigate the neurocognitive side effects by reducing the dose to the hippocampus. Recently, results of the phase II Radiation Therapy Oncology Group (RTOG) 0933 study showed evidence of improvements in neurocognitive outcomes compared to conventional WBRT for patients with multiple brain metastases (7). Nevertheless, the studies employing HA-WBRT generally exclude the region within 5 mm of the hippocampus, which may pose the risk of diminishing the clinical benefit of WBRT if the metastases are located in the spared region. Ghia et al. reported that the incidence of metastases within 5 mm of the hippocampus was very low (3.3%), which shows that the risks of HA-WBRT may be overestimated (8).

Intensity modulated radiation therapy (IMRT) has been used as a practical delivery method for HA-WBRT based on RTOG 0933 guidelines, whereas VMAT technique, which is based on simultaneous optimization of multi-leaf collimator (MLC) shapes, dose rate, and gantry rotation speed to achieve the desired dose distribution, have shown superior dosimetric performance and shorter treatment time compared with conventional IMRT (9–11). Despite these efforts, recent survey results indicated relatively low rates of utilization of HA-WBRT, which may result from the complexity of the treatment planning process owing to the anatomical shape and location of the hippocampus, the lack of dosimetry and physics support, and the suitability of patients.

To further improve the delivery efficiency, flattening-filter-free (FFF) technique has become increasingly popular due to its higher dose rates. As a result, the treatment delivery time can be reduced greatly, which is essential in improving patient comfort and limiting uncertainty of delivered dose related to intra-fraction motion (12). Hence, the FFF technique is particularly appealing for delivering stereotactic radiotherapy and has demonstrated great advantages due to significantly reducing treatment time without compromising the target coverage and organs at risk sparing (13–16). Furthermore, the removal of flattening filter is shown to have lower out-of-field dose on account of the diminution of head scatter and residual electron contamination in comparison to flattening-filtered (FF) mode (17). Patients may benefit from reduced exposure of healthy tissue to scattered doses outside the X-ray field. Dosimetric benefits of FFF also include less penumbra and MLC leakage (18). Therefore, FFF beams may offer a better solution in sparing the hippocampus and other OARs for HA-WBRT, in the hope of improving neurocognitive function impairment. With the advent of clinical FFF-linac, the planning studies of FFF mode have been carried out in various common cancer sites, such as prostate (19), hypopharynx (20), and breast (21). However, to the best of our knowledge, no investigation has been previously implemented for the dosimetric and radiobiological comparison of HA-WBRT using VMAT and IMRT with and without flattening filter.

The aim of this present study is to provide the first systematic clinical information on the application of the FFF irradiation mode in whole brain radiation therapy with hippocampal avoidance and evaluate whether the FFF mode is feasible and advantageous with respect to plan quality, delivery time and normal tissue complication probability (NTCP) for impaired neurocognitive function as compared to the flattening filter irradiation mode in VMAT and IMRT. Furthermore, considering the special anatomical location of the hippocampus, as well as the correlation between the performance of the FFF technique and the target volume, the impacts of hippocampal and target volumes on the differences between FFF beams and FF beams are also discussed.

## 2 Materials and methods

### 2.1 CT simulation and treatment preparation

With approval from our institutional review board, a total of 25 patients, who were diagnosed with brain metastases and underwent whole-brain radiation therapy with hippocampal avoidance at the Affiliated Hospital of Hebei University, were included and replanned in this retrospective study. The median age of the patients was 63 years (range: 37-76). Non-contrast computed tomography (CT) images for planning were obtained for all patients positioned supine and immobilized by means of a thermoplastic body mask using a large aperture 16 rows spiral CT of GE Medical System with 2.5 mm slice thickness. The DICOM images were then electronically sent to the Eclipse treatment planning system (Varian Medical Systems, Palo Alto, CA).

A gadolinium-enhanced, T1-weighted, three-dimensional spoiled gradient echo axial Magnetic Resonance Imaging (MRI) was acquired using a 1.5-T magnetic resonance scanner (Siemens AG, Munich, Germany) with 1.5 mm slice thickness for each patient. CT and MRI were rigidly co-registered by using an Eclipse mutual information algorithm. The hippocampus was manually contoured by an experienced radiation oncologist using the RTOG 0933 atlas as reference. According to the volume of the hippocampi (range: 1.12 cm^3^ - 4.59 cm^3^), the patients were divided into three groups for subsequent volume-based analysis, as Group 1 (1 cm^3^< hippocampi ≤ 2 cm^3^), Group 2 (2 cm^3^< hippocampi ≤ 3 cm^3^), Group 3 (3 cm^3^< hippocampi ≤ 4.59 cm^3^). The hippocampal avoidance zone (HAZ) was created using a 5-mm volumetric expansion of the hippocampi to account for necessary dose fall-off between the hippocampi and the target. The following volumes of interest were also delineated: clinical target volume (CTV, CTV was defined as the whole brain parenchyma), lens, optic nerve and optic chiasm. The planning target volume (PTV) was constructed by expanding the CTV by 3 mm in all directions. The planning target volume with hippocampal avoidance (PTV-HA) used for dose optimization was generated by subtracting the HAZ from PTV. For the other grouping method, all patients were stratified in three groups according to the volume of PTV-HA (range: 1098.3 cm^3^ - 2056.8 cm^3^): Group 4 (1000 cm^3^< PTV-HA ≤ 1568.2 cm^3^), Group 5 (1568.2 cm^3^< PTV-HA ≤ 1867.9 cm3), Group 6 (1867.9 cm^3^< PTV-HA ≤ 2056.8 cm^3^).

### 2.2 Linear accelerator

The treatment planning was implemented using Varian Trilogy linear accelerator (Varian Medical Systems, Palo Alto, CA, USA), which has the flattened as well as unflattened beams and is equipped with 120 Millennium multi-leaf collimator leaves. The leaf width is 5 mm in the central 20-cm part of the field and 10 mm in the outer 2×10 cm. Removing the flattening filter from the beam path increases the dose rate up to 1400 MU/min for 6 MV but decreases the beam quality index (TPR20/10: 6 MV 0.669, 6 MV(FFF) 0.629).

### 2.3 Treatment planning setup

Four different plans were optimized for each patient based on Eclipse treatment planning system using intensity-modulated radiotherapy and volumetric modulated arc therapy with 6 MV photon beams with flattening filter or without, in the following referred to as IMRT-FF, IMRT-FFF, VMAT-FF and VMAT-FFF plans. The maximum dose rate was adopted to leave the highest degree of freedom in the optimization process, which was 600 MU/min for FF beams, and 1400 MU/min for FFF beams. For IMRT, the dose rate is always maintained at the maximum during dose delivery. However, due to mechanical motion speed restrictions, the maximum dose rate will not be applied throughout the VMAT process. The total dose prescribed was 30 Gy delivered in 10 fractions to the PTV-HA for all the studied cases. The IMRT plans were realized by sliding window dynamic delivery method and consisted of seven equispaced beams with gantry angles of 204°, 256°, 308°, 0°, 52°, 104° and 156°. The collimator was angled to 0° in IMRT plans. To reduce treatment time and the possibility of operating errors, the couch angles of all fields were set to 0° instead of the noncoplanar beam arrangement recommended by RTOG. The VMAT plans were generated using RapidArc technique with two coplanar arcs as clockwise arc 181°–179° and anti-clockwise arc 179°–181°. Gantry spacing between two control points was 4°. In addition, to reduce the MLC tongue-and-groove leaves’ leakage, the collimator angle was set to 30 degrees for the first clockwise arc and the collimator of the second anti-clockwise arc was 330 degrees (22). For all plans, the isocenter was located centrally in the PTV-HA based on beam’s eye view graphic. All plans utilized the Photon Optimizer (Version 13.6.23, Varian, Palo Alto, CA, USA) to optimize the intensity map for IMRT and determine the optimal combination of beam weight and shape for VMAT. The constraints for target and OARs were matched to RTOG 0933 planning requirements (see **Table 1** for the RTOG criteria). Identical dose volume objectives and weights were used for optimization of four plans for each patient to make the results comparable. The Analytical Anisotropic Algorithm (AAA) along with a grid resolution of 2.5 mm and heterogeneous corrections were adopted to arrive at dose calculations.

**Table 1:** Dosimetric compliance criteria for hippocampal sparing in RTOG 0933.

Parameter	Dose constraints
PTV-HA	D_2%_ ≤ 37.5 Gy (D_2%_ ≤ 40 Gy is allowed) D_98%_ ≥ 25 Gy and V_30_ ≥ 90%
Hippocampus	D_100%_ ≤ 9 Gy (D_100%_ ≤ 10 Gy is allowed) D_max_ ≤ 16 Gy (D_max_ ≤ 17 Gy is allowed)
Optic chiasm	D_max_ ≤ 37.5 Gy
Optic nerves	D_max_ ≤ 37.5 Gy

### 2.4 Plan comparison

Quantitative evaluation of the plans was performed by analysis of the dose-volume histograms (DVHs) extracted from the planning system with respect to target coverage, dose homogeneity and conformity, and OAR sparing. For the purpose of comparison, all plans were normalized to meet the same objectives with the 95% of the PTV-HA volume surrounded by the 100% isodose line. The dose distribution of the target was evaluated in terms of mean dose, D_2%_ and D_98%_ (dose received by 2% and 98% of target volume), conformity index (CI), prescription isodose/target volume (PITV) ratio, and homogeneity index (HI). CI was calculated using the equation:


(1) $$CI=V_{t,ref}^{2}/(V_{t}\cdot V_{ref})$$

according to Paddick (23). Here V_t,ref_ represented the volume receiving a dose equal to or greater than the reference dose in the target volume, V_t_ stood for the target volume, and V_ref_ was the total volume covered by a dose equal to or greater than the reference dose. The reference dose was the 95% of the prescription dose in this study. CI ranged from 0 to 1, and the higher the CI, the better the conformity of the target volume. For comparison and reference purposes, dose conformity was also quantified using PITV, defined as the prescription isodose volume divided by the target volume. Since target coverage was maintained at 95% in this study, the smaller PITV indicated better conformity and less radiation exposure to normal tissue. HI was defined as follows:


(2) $$\text{HI}=(D_{2\%}-D_{98\%})/D_{prescription}$$

where D_prescription_ meant the prescription dose. The ideal value of HI was 0, which indicated a sharp dose fall between the neck region and tail region of the PTV-HA dose-volume histogram, with increasing values for the metric indicative of declining homogeneity throughout the volume.

Concerning the hippocampus, we considered the maximum, mean, and quintile (D_20%_ to D_100%_) doses to assess hippocampal sparing. The maximum doses of the lens, optic chiasm, and optic nerve were also reported. Moreover, an additional structure called non-tumor tissue (NT) consisting of body minus PTV was created. The integral dose (24) for non-tumor tissue was calculated according to the following formula as a measure of low dose in the periphery: Integral dose = Mean dose (Gy) × Volume (cm^3^). Furthermore, the number of monitor units (MUs) required per fraction dose for the four techniques was also compared.

### 2.5 Quality assurance

Dose verifications were performed using the ArcCheck Phantom (Sun Nuclear Corporation, Melbourne, USA) to ensure the deliverability of each treatment plan. It is a cylindrical water-equivalent phantom for patient specific quality assurance (QA) with a three-dimensional array of 1386 diode detectors. The measured doses at the detectors plane were compared with the predicted dose distribution previously calculated in Eclipse planning system. Evaluation was based on gamma analysis by SNC patient software with criteria of 3% maximum dose difference and 2 mm distance to agreement as recommended by the AAPM TG 218 (25). A global normalization for the absolute dose was performed. The agreement between the measured dose distribution and calculated dose distribution was considered acceptable if the gamma indexes of at least 95% of the pixels with a dose value of ≥ 10% of the maximum dose were smaller than 1. The treatment delivery time was documented from first beam on to last beam off when the QA plan was delivered. The mean dose rates of VMAT in both delivery modes were also calculated. In addition, the measured data of all QA plans were collected by delivering at the machine in one session to minimize the impact of machine output rate on QA results.

### 2.6 Radiobiological indices

Biologically equivalent dose in 2-Gy fractions (EQD_2_) to 40% of the bilateral hippocampi was computed using a hippocampal α/β value of 2 (26). The NTCP for neurocognitive function impairment of the hippocampus was assessed for all plans according to the model proposed by Gondi et al. (26). The model was based on the Lyman model and its formula was expressed as follows:


(3) $$\text{NTCP}=\frac{1}{\sqrt{2\pi }}\int_{-\infty }^{t}\exp(-u^{2}/2)du$$

where,


(4) $$t=\frac{EQD_{2}(D_{40\%})-TD_{50}}{mTD_{50}}$$

EQD_2_(D_40%_) was EQD_2_ received by 40% of bilateral hippocampal volume, TD_50_ was the EQD_2_(D_40%_) value corresponding to a 50% probability of neurocognitive decline, and m represented the slope of the dose-response curve. Moreover, TD_50_ and m were estimated to be 14.88 Gy and 0.54 by Gondi et al. (26).

### 2.7 Statistical evaluation

All data were reported as mean and standard deviation. The Wilcoxon matched-pair signed-rank test, a non-parametric test, implemented in SPSS software version 22 (SPSS, IBM Corp, Armonk, NY), was used for statistical analysis, and the difference was considered statistically significant when p< 0.05.

## 3 Results

**Figure 1:**
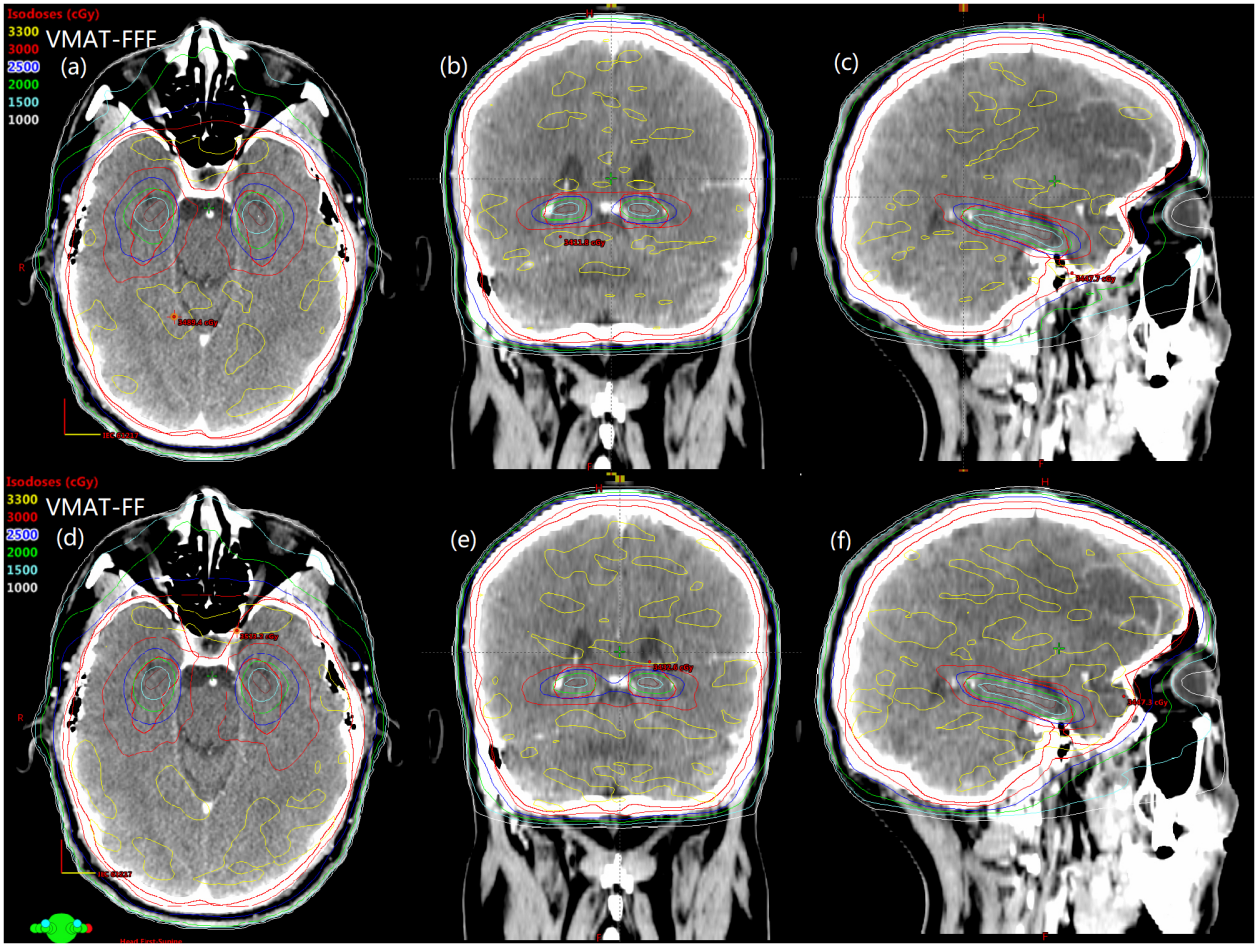
The comparison of spatial isodose distributions of VMAT-FFF **(A–C)** versus VMAT-FF **(D–F)** for a sample patient. Red contour represents the PTV-HA and brown contour represents the hippocampus.

**Figure 2:**
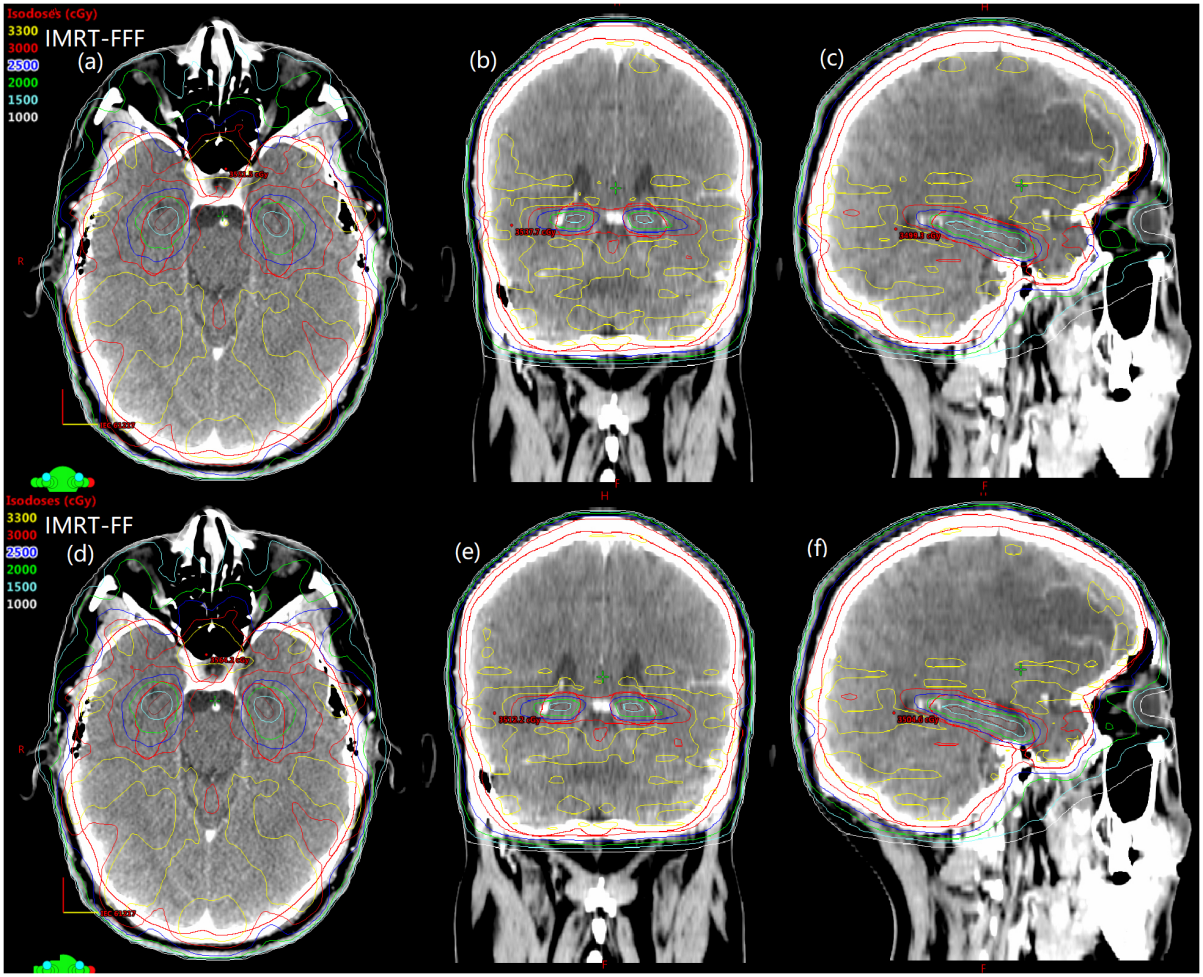
Spatial dose distributions in axial, coronal, sagittal views for one representative patient with avoidance of hippocampus during whole brain radiotherapy using IMRT-FFF **(A–C)** and IMRT-FF **(D–F)** techniques. PTV-HA and hippocampus are drawn in red and brown, respectively.

**Figure 3:**
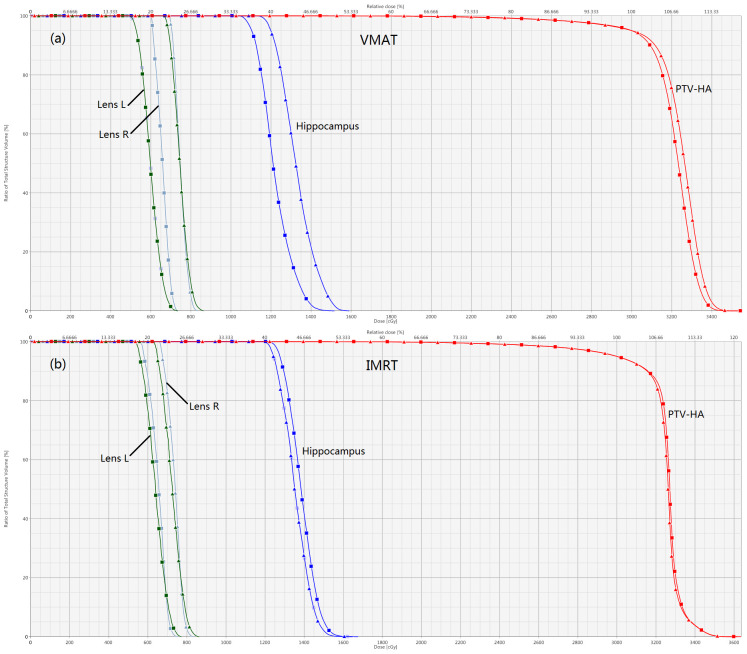
Normalized dose-volume histograms for a sample hippocampal-sparing WBRT patient. Comparisons of FFF versus FF for VMAT and IMRT are displayed in **(A, B)**, respectively. The lines with squares represent FFF plans, and the lines with triangles stand for FF plans. The optic chiasm and optic nerve are not shown for the sake of clarity.

Details about DVH parameters averaged over all patients with regard to target coverage and OAR sparing are summarized in **Table 2** for the comparison of the two irradiation modes FF and FFF. As to four kinds of plans for a representative patient, the spatial isodose distributions with display of an axial, sagittal and coronal plane at the level of hippocampus are presented in **Figures 1** and **2**, and the DVHs involving the PTV-HA, hippocampus and lens are showed in **Figure 3**.

**Table 2:** Comparison of dose distributions of PTV-HA and OARs for VMAT and IMRT with the two irradiation modes FFF and FF.

Variable	VMAT-FFF	VMAT-FF	p^a^	IMRT-FFF	IMRT-FF	p^b^
PTV-HA						
D_2%_	33.24 ± 0.44	33.66 ± 0.52	<0.001	34.62 ± 0.57	34.57 ± 0.57	0.001
D_98%_	27.11 ± 0.39	26.81 ± 0.40	<0.001	27.30 ± 0.29	27.31 ± 0.31	0.074
D_mean_	31.74 ± 0.29	32.11 ± 0.38	<0.001	32.64 ± 0.36	32.54 ± 0.36	<0.001
HI	0.19 ± 0.02	0.21 ± 0.02	<0.001	0.22 ± 0.02	0.22 ± 0.02	0.016
CI	0.87 ± 0.02	0.85 ± 0.02	<0.001	0.83 ± 0.02	0.83 ± 0.02	0.326
PITV	1.04 ± 0.02	1.07 ± 0.02	<0.001	1.10 ± 0.02	1.10 ± 0.02	0.459
Hippocampus						
D_20%_	12.07 ± 0.58	12.93 ± 0.73	<0.001	14.38 ± 0.38	14.09 ± 0.38	<0.001
D_40%_	11.46 ± 0.57	12.28 ± 0.72	<0.001	13.89 ± 0.35	13.61 ± 0.35	<0.001
D_60%_	10.96 ± 0.54	11.73 ± 0.68	<0.001	13.50 ± 0.35	13.23 ± 0.34	<0.001
D_80%_	10.45 ± 0.50	11.16 ± 0.64	<0.001	13.09 ± 0.37	12.83 ± 0.34	<0.001
D_100%_	9.37 ± 0.39	9.98 ± 0.56	<0.001	12.00 ± 0.39	11.87 ± 0.33	0.01
D_mean_	11.28 ± 0.51	12.07 ± 0.65	<0.001	13.75 ± 0.34	13.48 ± 0.34	<0.001
D_max_	15.14 ± 0.50	16.15 ± 0.58	<0.001	17.51 ± 1.07	17.18 ± 1.03	<0.001
Lens L						
D_max_	6.91 ± 0.62	8.02 ± 0.73	<0.001	7.80 ± 0.64	8.43 ± 0.74	<0.001
Lens R						
D_max_	6.95 ± 0.56	8.02 ± 0.71	<0.001	8.02 ± 0.94	8.49 ± 0.95	<0.001
Optic chiasm						
D_max_	33.79 ± 0.68	34.19 ± 0.64	0.009	35.37 ± 0.55	35.24 ± 0.59	0.011
Optic nerve L						
D_max_	31.47 ± 5.94	33.22 ± 1.11	0.002	32.79 ± 2.57	32.88 ± 2.54	0.019
Optic nerve R						
D_max_	32.70 ± 1.14	33.28 ± 1.08	0.001	33.19 ± 2.41	33.30 ± 2.28	0.162
NT						
Integral dose	31.55 ± 3.97	32.42 ± 4.02	<0.001	32.04 ± 4.25	32.46 ± 4.42	<0.001

The results of Wilcoxon matched-pair signed-rank test are also listed.

Dose values are given in Gy. Integral dose is given in Gy*cm^3^*10^3^. ^a^ p value denotes the results of Wilcoxon test between VMAT-FFF and VMAT-FF plans. ^b^ p value means the results of Wilcoxon test between IMRT-FFF and IMRT-FF plans.

### 3.1 Target coverage

It could be seen in **Table 2** that the D_2%,_ D_98%_, D_mean_, HI, CI, and PITV were improved significantly for the VMAT planning techniques if flattening filter free beams were used, and the corresponding p values were all less than 0.001. On the contrary, the dose volume parameters of IMRT-FF plans were either slightly better or not significantly different compared to IMRT-FFF plans. It was worth noting that the removal of flattening filter had a greater impact on VMAT plans than IMRT. In addition, VMAT plans were remarkably superior compared to IMRT plans.

### 3.2 OARs sparing

The maximum and minimum doses of hippocampus for VMAT-FFF were 15.14 ± 0.50 Gy and 9.37 ± 0.39 Gy, which were significantly lower than the corresponding values of VMAT-FF, and the improvement of dose sparing for all the evaluation indicators involving the hippocampus in the VMAT-FFF was statistically significant compared with VMAT-FF. Dose constraints to hippocampus were reached for VMAT with or without the use of the FF, while the cumulative averages of D_max_ and D_100%_ for hippocampus were 17.51 ± 1.07 Gy and 12.00 ± 0.39 Gy, 17.18 ± 1.03 Gy and 11.87 ± 0.33 Gy for IMRT-FFF and IMRT-FF, respectively, which did not meet the requirements for hippocampal sparing according to RTOG 0933 guidelines. For IMRT, the FF mode showed better results for hippocampus compared to the FFF, while the difference between the treatment modes was generally only about 2% even though statistically significant.

In addition to hippocampal sparing, the FFF technique also obviously reduced the doses to other OARs such as lens, optic chiasma, and optic nerve compared to FF mode with respect to VMAT (all p< 0.05). The maximum doses to left and right lenses in the VMAT-FFF plans on average were 6.91 ± 0.62 Gy and 6.95 ± 0.56 Gy, which were 13.8% and 13.3% lower than the corresponding values of VMAT-FF. A similar result can be found in the IMRT plans for lens. Concerning optic chiasma and optic nerve, D_max_ was rather close between FF and FFF for IMRT. As for non-tumor tissue, FFF beams showed lower integral dose for both VMAT and IMRT compared with conventional beams.

In a word, the VMAT plans using FFF beams improved the homogeneity and conformity of the target and reduced OARs doses significantly keeping target coverage at the same level in comparison with the other three planning techniques.

### 3.3 NTCP for neurocognitive function impairment

**Table 3** shows the computed NTCP for neurocognitive function impairment for the FFF and FF plans. The NTCP of VMAT-FFF plans was significantly lower than that of VMAT-FF plans. The opposite result was seen for IMRT technique, however the difference between FFF and FF plans was small.

**Table 3:** Comparison of EQD_2_(D_40%_) and NTCP for four types of plans.

Parameters	VMAT-FFF	VMAT-FF	p^a^	IMRT-FFF	IMRT-FF	p^b^
EQD_2_(D_40%_)	9.02 ± 0.61	9.92 ± 0.8	<0.001	11.78 ± 0.41	11.44 ± 0.41	<0.001
NTCP	0.23 ± 0.02	0.27 ± 0.03	<0.001	0.35 ± 0.02	0.34 ± 0.02	<0.001

Dose values are given in Gy. ^a^ p value denotes the results of Wilcoxon test between VMAT-FFF and VMAT-FF plans. ^b^ p value means the results of Wilcoxon test between IMRT-FFF and IMRT-FF plans.

### 3.4 Plan verification and efficiency

The monitor units, treatment time and passing rate of γ for each treatment modality, and the mean dose rates of VMAT plans are listed in **Table 4**. Meanwhile, **Table 4** also shows the results of the Wilcoxon statistical test for between VMAT-FFF and VMAT-FF as well as between IMRT-FFF and IMRT-FF. The number of monitor units employed was dramatically reduced by 81.5% for VMAT-FFF compared with IMRT-FFF and 79.2% for VMAT-FF compared with IMRT-FF. Furthermore, FFF plans required more MUs than FF plans, with an increase of 19.2% for VMAT and 33.8% for IMRT. The mean treatment time was reduced by 26% for IMRT in FFF mode as compared to FF, but was almost the same in both irradiation modes in case of VMAT. Besides, VMAT took less treatment time than IMRT technique. As shown in **Table 4**, VMAT-FFF provided a higher mean dose rate than VMAT-FF, but both types of VMAT plans were well below their respective maximum dose rates. The analyzed data indicated that all 100 plans were clinically deliverable and passed the gamma evaluation. The gamma indices did not show any notable distinctions between FFF and FF for both VMAT and IMRT.

**Table 4:** Comparison of monitor units, treatment time, mean dose rate and the results of gamma analysis for four types of plans.

Variable	VMAT-FFF	VMAT-FF	p^a^	IMRT-FFF	IMRT-FF	p^b^
Monitor units	844 ± 35	708 ± 35	<0.001	4561 ± 263	3409 ± 197	<0.001
Treatment time (min)	3.10 ± 0.02	3.12 ± 0.02	0.56	6.90 ± 0.33	9.32 ± 0.46	<0.001
Mean dose rate (MU/min)	337.6 ± 14.1	283.0 ± 13.9	<0.001	**-**	**-**	**-**
Passing rate of γ	99.7 ± 0.4	99.8 ± 0.3	0.67	97.9 ± 0.7	97.6 ± 1.1	0.48

^a^ p value denotes the results of Wilcoxon test between VMAT-FFF and VMAT-FF plans. ^b^ p value means the results of Wilcoxon test between IMRT-FFF and IMRT-FF plans.

### 3.5 A volume-based analysis

**Figure 4:**
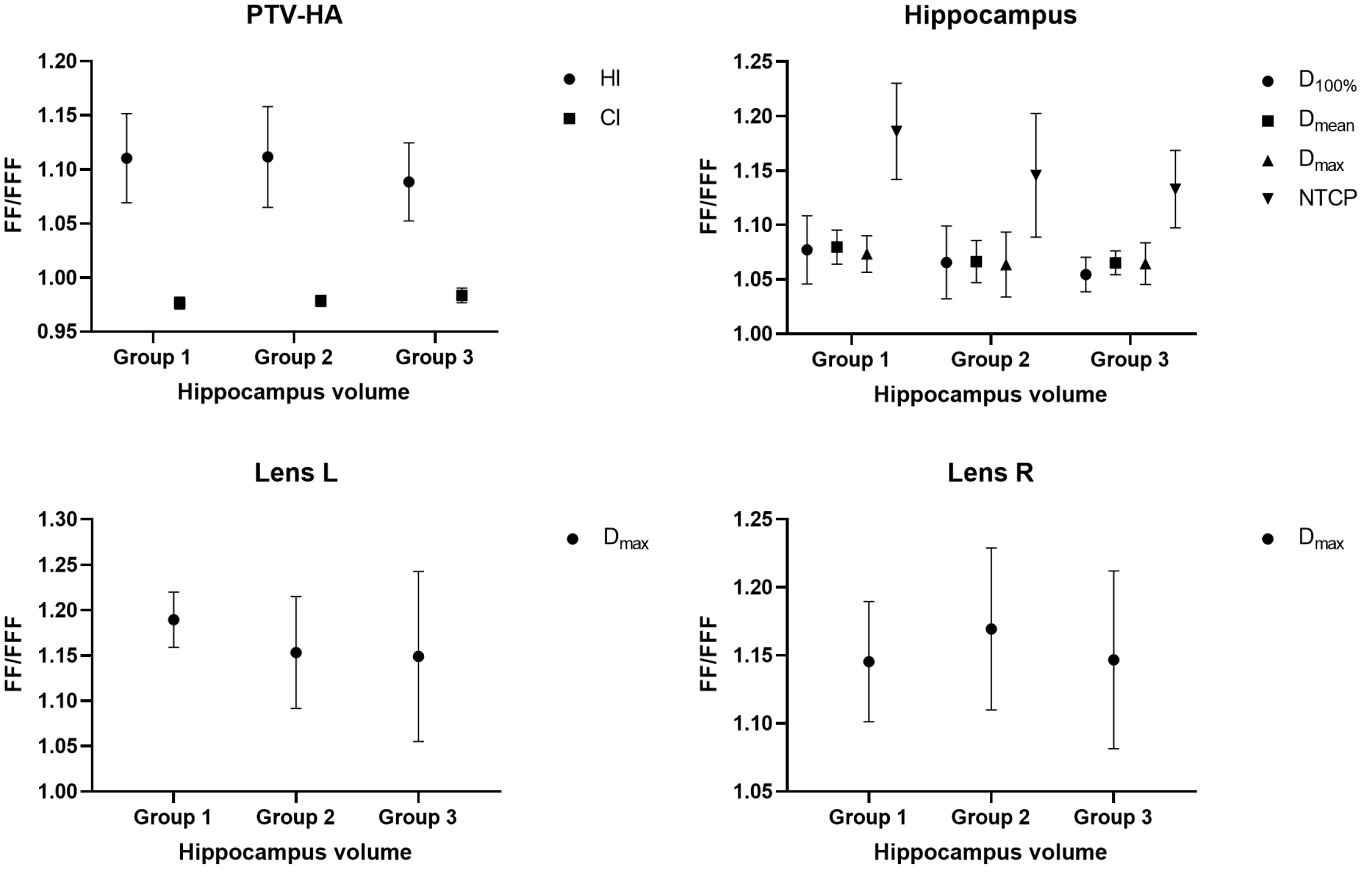
Ratio of FF and FFF plans for parameters of PTV-HA, hippocampus and lens plotted against hippocampal volume. All patients were stratified into three groups according to the hippocampal volume.

**Figure 5:**
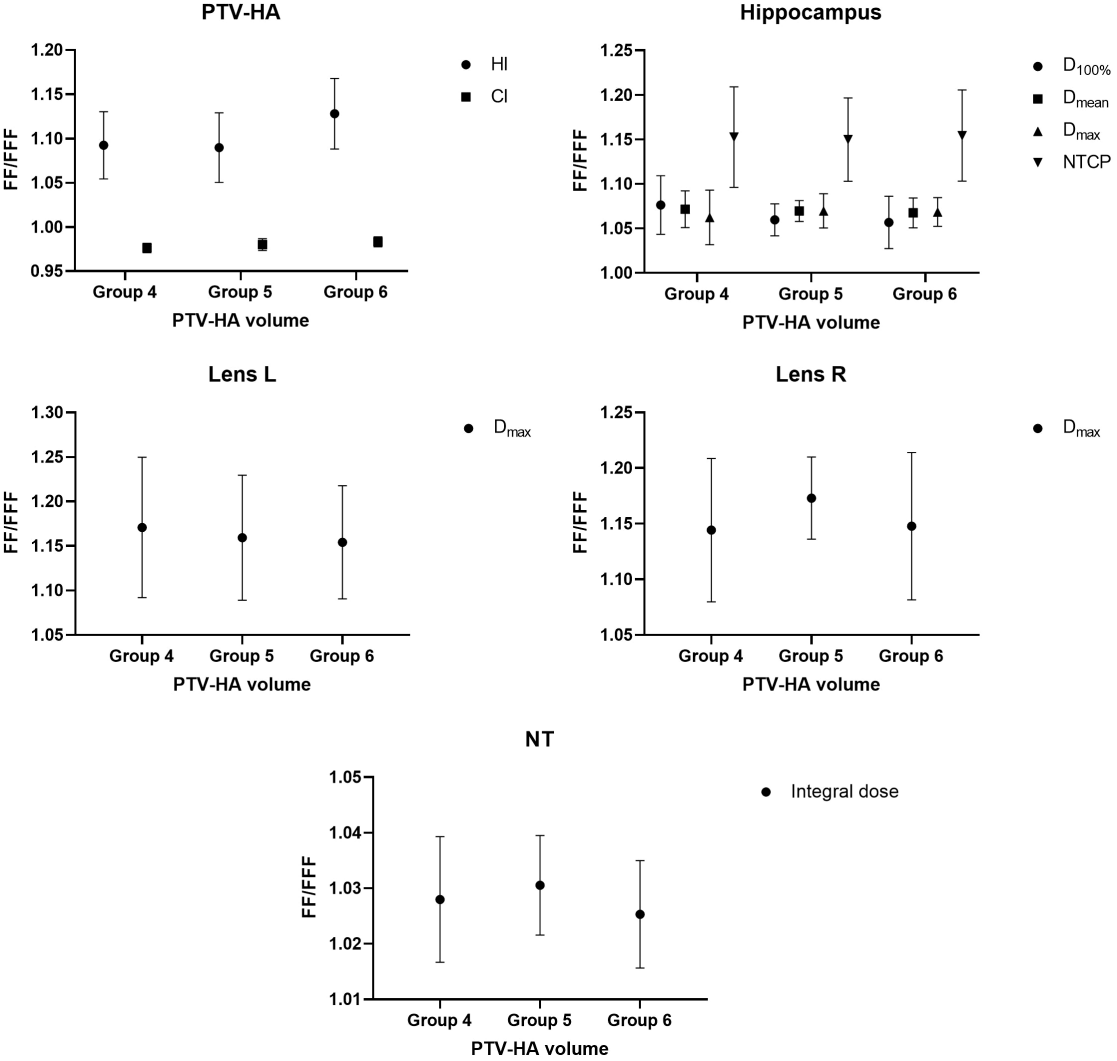
Ratio of FF and FFF plans for parameters of PTV-HA, hippocampus, lens and NT plotted against PTV-HA volume. All patients were stratified into three groups according to the PTV-HA volume.

The research results above showed that VMAT achieved significantly better plan quality than IMRT. There was little difference between FF and FFF plans for IMRT. Thus, the volume-based analysis was performed only for the VMAT technique. All values for PTV-HA (HI and CI), the hippocampus (D_100%_, D_mean_, D_max_ and NTCP), Lens L (D_max_) and Lens R (D_max_) in the three volume-dependent groups are provided in **Table 5** for grouping according to hippocampal volume and **Table 6** for grouping according to PTV-HA volume. The FF/FFF ratio was computed for all the parameters described above in the matched plans (e.g., HI_VMAT-FF_/HI_VMAT-FFF_). Then, the FF/FFF fraction for each group is plotted as a function of the corresponding volume, as shown in **Figure 4** for grouping according to hippocampal volume and **Figure 5** for grouping according to PTV-HA volume. In addition, the integral dose of NT is also considered for grouping based on PTV-HA volume. There is no significant trend in other dosimetric parameters with hippocampal and PTV-HA volumes, and the results are not shown.

**Table 5:** Summary of the results for parameters of PTV-HA, hippocampus and lens in three groups according to the hippocampal volume.

	Parameters		Group 1	Group 2	Group 3
PTV-HA	HI	VMAT-FFF	0.19 ± 0.02	0.20 ± 0.02	0.19 ± 0.03
		VMAT-FF	0.21 ± 0.02	0.22 ± 0.02	0.21 ± 0.03
		p	0.018	0.008	0.008
	CI	VMAT-FFF	0.87 ± 0.02	0.86 ± 0.02	0.87 ± 0.01
		VMAT-FF	0.85 ± 0.03	0.84 ± 0.02	0.85 ± 0.02
		p	0.018	0.008	0.008
Hippocampus	D_100%_	VMAT-FFF	9.71 ± 0.39	9.34 ± 0.22	9.13 ± 0.36
		VMAT-FF	10.46 ± 0.60	9.95 ± 0.45	9.62 ± 0.33
		p	0.018	0.008	0.008
	D_mean_	VMAT-FFF	11.70 ± 0.45	11.31 ± 0.44	10.93 ± 0.38
		VMAT-FF	12.63 ± 0.59	12.06 ± 0.62	11.64 ± 0.38
		p	0.018	0.008	0.008
	D_max_	VMAT-FFF	14.83 ± 0.57	15.13 ± 0.49	15.40 ± 0.34
		VMAT-FF	15.91 ± 0.57	16.09 ± 0.69	16.39 ± 0.43
		p	0.018	0.008	0.008
	NTCP	VMAT-FFF	0.25 ± 0.02	0.23 ± 0.02	0.22 ± 0.02
		VMAT-FF	0.30 ± 0.03	0.27 ± 0.03	0.25 ± 0.02
		p	0.017	0.007	0.007
Lens L	D_max_	VMAT-FFF	6.60 ± 0.79	6.96 ± 0.42	7.10 ± 0.60
		VMAT-FF	7.85 ± 0.96	8.02 ± 0.51	8.14 ± 0.79
		p	0.018	0.008	0.008
Lens R	D_max_	VMAT-FFF	6.71 ± 0.70	6.96 ± 0.40	7.11 ± 0.56
		VMAT-FF	7.69 ± 0.83	8.13 ± 0.41	8.16 ± 0.83
		p	0.018	0.008	0.008

Dose values are given in Gy. p value denotes the results of Wilcoxon test between VMAT-FFF and VMAT-FF plans.

**Table 6:** Summary of the results for parameters of PTV-HA, hippocampus, lens and NT in three groups according to the PTV-HA volume.

	Parameters		Group 4	Group 5	Group 6
PTV-HA	HI	VMAT-FFF	0.19 ± 0.02	0.19 ± 0.01	0.19 ± 0.03
		VMAT-FF	0.21 ± 0.02	0.21 ± 0.02	0.21 ± 0.03
		P	0.008	0.012	0.012
	CI	VMAT-FFF	0.85 ± 0.01	0.87 ± 0.01	0.88 ± 0.01
		VMAT-FF	0.83 ± 0.01	0.85 ± 0.01	0.87 ± 0.01
		p	0.008	0.012	0.012
Hippocampus	D_100%_	VMAT-FFF	9.28 ± 0.22	9.35 ± 0.58	9.47 ± 0.33
		VMAT-FF	9.99 ± 0.52	9.91 ± 0.67	10.02 ± 0.55
		p	0.008	0.012	0.012
	D_mean_	VMAT-FFF	11.20 ± 0.39	11.21 ± 0.74	11.45 ± 0.34
		VMAT-FF	12.00 ± 0.61	11.99 ± 0.83	12.22 ± 0.55
		p	0.008	0.012	0.012
	D_max_	VMAT-FFF	15.10 ± 0.56	15.32 ± 0.40	15.02 ± 0.54
		VMAT-FF	16.04 ± 0.72	16.38 ± 0.45	16.05 ± 0.54
		p	0.008	0.012	0.012
	NTCP	VMAT-FFF	0.23 ± 0.02	0.23 ± 0.03	0.24 ± 0.02
		VMAT-FF	0.27 ± 0.03	0.27 ± 0.04	0.28 ± 0.03
		p	0.007	0.011	0.011
Lens L	D_max_	VMAT-FFF	6.60 ± 0.61	7.32 ± 0.21	6.84 ± 0.72
		VMAT-FF	7.72 ± 0.72	8.48 ± 0.43	7.89 ± 0.82
		P	0.008	0.012	0.012
Lens R	D_max_	VMAT-FFF	6.74 ± 0.57	7.25 ± 0.32	6.87 ± 0.64
		VMAT-FF	7.70 ± 0.65	8.51 ± 0.42	7.88 ± 0.80
		p	0.008	0.012	0.012
NT	Integral dose	VMAT-FFF	29.84 ± 3.04	32.50 ± 4.73	32.52 ± 3.88
		VMAT-FF	30.65 ± 2.89	33.49 ± 4.88	33.34 ± 3.97
		p	0.008	0.012	0.012

Dose values are given in Gy. p value denotes the results of Wilcoxon test between VMAT-FFF and VMAT-FF plans.

There was a tendency for the conformity and homogeneity of PTV-HA to worsen and then improve with the increase in hippocampal volume. The homogeneity remained stable with increasing PTV-HA volume, while the conformity became better. The differences between FF and FFF for PTV-HA were larger in Group 1 and Group 6. With the increase in hippocampal volume, the D_100%_, D_mean_ and NTCP of hippocampus gradually decreased, while D_max_ gradually increased. All indices of the hippocampus showed little change among different PTV-HA volumes. The biggest difference between the two delivery modes was observed in Group 1 for all the evaluation parameters of the hippocampus when grouping was determined by hippocampal volume. However, the fluctuations of the FF/FFF values for hippocampus were not noticeable with changes in target volume. The lens had the lowest D_max_ in cases with small hippocampal and target volumes. The benefit of FFF was slightly greater for small hippocampal and target volumes in terms of the left lens. However, the advantage of FFF was greatest for medium hippocampal and target volumes in terms of the right lens. With the increase in target volume, the integral dose of NT also significantly increased, but the range of variation in the FF/FFF ratio was small.

## 4 Discussion

The flattening filter free delivery mode of a linear accelerator is not a new idea in radiation therapy but it has only recently become a reality for clinical routine. In this study, we presented the first evaluation of the potential of the flattening filter free mode in intensity modulated radiation therapy and volumetric modulated arc therapy for patients with hippocampal avoidance whole brain radiotherapy. In terms of target homogeneity and conformity, and OAR sparing, the VMAT-FFF has shown superior quality compared to the VMAT-FF, which can potentially reduce radiation induced inflammation in the hippocampus and its associated neurologic functional sequelae. For IMRT in this article, FFF beams led to similar plan quality compared to FF. IMRT-FFF had a lower dose to the lens while IMRT-FF achieved better hippocampal protection. Obviously, FFF mode is feasible and beneficial in whole brain radiotherapy with hippocampal avoidance, especially for VMAT. A planning study of right sided breast cancer indicated that VMAT-FFF achieved better target coverage and homogeneity than VMAT-FF with similar doses to the OARs while IMRT-FF showed better results regarding some parameters of OARs without compromising target coverage and homogeneity compared to IMRT-FFF (27). For gastric cancers (28) and patients with in-field recurrence of vertebral metastases (29), FFF plans significantly reduced the dose to the normal tissue, while maintaining target coverage, conformity and homogeneity comparable to FF plans for both IMRT and VMAT. However, differences in plan quality were insignificant between the two irradiation modes for carcinoma of the hypopharynx/larynx (20). It was worth mentioning that, for large and complex targets, such as advanced nasopharyngeal carcinoma (30), VMAT-FFF showed poorer conformity and homogeneity of the target compared to VMAT with traditional flattened beam, and VMAT-FF was more likely to result in a lower dose for most OARs. Based on previous researches, it can be concluded that it is not possible to generalize the results of planning studies for a specific combination of equipment and tumor site, and different targets must be investigated individually.

Mounting evidence imputes neurocognitive deficits in learning and memory after conventional WBRT to radiation induced inflammatory to the neural stem cell compartment in the hippocampus. Hence, the avoidance of hippocampus in the course of WBRT treatments has been proposed to achieve prospective neurocognitive benefits, and continued researches have been placed on this area. Using helical tomotherapy and linear accelerator-based IMRT technique, Gondi et al. have reported their preliminary experience and have presented excellent results with hippocampal-sparing whole-brain radiotherapy for patients with brain metastases (31). The hippocampus was spared by helical tomotherapy, which was administered at a median dose of 5.5 Gy and a maximum dose of 12.8 Gy. The hippocampus was spared by noncoplanar IMRT based on linac, resulting in a median dose of 7.8 Gy and a maximum dose of 15.3 Gy. It has previously been reported that the mean and maximum doses to hippocampus respectively were 11.2 ± 0.3 Gy, and 15.6 ± 0.4 Gy with 90.5% of the target volume surrounded by the prescription dose isoline, exhibited by intensity-modulated arc therapy approach for whole brain radiotherapy patients with sparing hippocampus (32). Volumetric modulated arc therapy plans with two full coplanar arcs generated by Auto-Planning engine offered 91.5% coverage for target and 16 Gy of the maximum dose to the hippocampus (33). For VMAT-FFF in this study, the maximum dose in the hippocampus was 15.14 ± 0.50 Gy, when the plans were established at 95% of the volume of PTV-HA to achieve 100% of the prescribed dose. Therefore, compared with previously published researches utilizing VMAT technique, the VMAT plans using FFF beams can attain comparable or lower dose to hippocampus with better target coverage.

Keeping the mean hippocampus dose below 12 Gy out of 30 Gy in 10 fractions prescription is recommended to improve neurocognitive function (34). The treatment plan created by VMAT-FF was very close to the recommended value. The hippocampus D_mean_ of VMAT-FFF plans was 11.28 ± 0.51 Gy, which was lower than the protocol requirement of 12 Gy. Nevertheless, neither IMRT-FFF nor IMRT-FF could satisfy the criteria.

Although some differences in the plan comparisons were statistically significant, the clinical relevance of these differences remains unclear. To address this issue to some extent, NTCP, an indication of the severity of damage to normal tissues, was calculated. The results of this study showed that VMAT-FFF had the lowest NTCP for impaired neurocognitive function, which was agreed well with the better sparing of hippocampus. It’s worth noting that the slight differences, such as the maximum doses to optic nerve and optic chiasm, may not be clinically significant. Hence, the practical benefits of using FFF beams have yet to be verified by clinical results.

Hippocampal volume had a large effect on the planning parameters, as shown in **Table 5**. For example, the treatment planning with the small hippocampal volume resulted in the better dose distribution of target and lower D_max_ values of hippocampus and lens. Therefore, accurate delineation of the hippocampus is necessary in order to achieve neuroprotective benefits. However, the hippocampus delineated varies greatly due to the differences in experience of radiation oncologist, quality of MRI, and the criteria for contouring of the hippocampus in different cancer centers. For instance, the volume of hippocampi was 2.68 ± 1.05 cm^3^ in this study, whereas the value was 3.30 cm^3^ described by Gondi et al. (31). Certainly, with the availability of the hippocampal atlas and continuing instruction, the delineation of hippocampus will become more accurate and uniform with time.

For VMAT, FFF beams achieved significantly better plan quality than FF beams over the entire range of hippocampal and target volumes. With the decrease in hippocampal volume, there will be an increase in the absolute difference between FFF and FF beams for the parameters of PTV-HA and the hippocampus. This finding can serve as a reference for selecting patients when utilizing the FFF technique for HA-WBRT. Nevertheless, the difference between FF and FFF seems to be insensitive to changes in target volume.

The analysis of the technical delivery parameters revealed that the number of MUs was higher for FFF compared to FF regardless of IMRT or VMAT, which was in line with these studies of advanced esophageal cancer (35) and prostate cancer (19). This effect is conceptually expected because the intensity of FFF beam decreases with the off-axis distance, which is evident in open beam dose profiles for larger fields (≥ 10 × 10 cm^2^). Consequently, at the periphery of the larger target, additional MUs are required to gain a uniform dose distribution on account of the unflattened profile of the FFF beam. In addition, FFF plans generally have more modulation owing to the capability for higher MUs and the inherent beam profile shape itself. Furthermore, the higher amount of MUs and dose rate of FFF plan have raised concerns about radiation protection. In fact, the removal of FF gives rise to a significant decrease in neutron fluence and dose equivalent within the treatment room (36). The photon dose at the maze door in FFF mode is always lower than the dose measured in FF mode, regardless of the presence or absence of a water phantom and the size of the field opening (37). The required thickness of primary barriers is reduced by 8% when unflattened beams are used (38). Hence, existing shielding is usually adequate and surplus if instead of the standard flattened photon beams unflattened ones are used, which can reduce occupational exposure for staff, assuming a constant permitted dose per week. Although, an increased number of MUs with FFF was observed for IMRT, the treatment time was cut by 2.42 minutes because of the higher dose rate, improving patient stability and treatment accuracy. The time advantage of using FFF beams increases with increasing dose per fraction, which makes FFF beams especially attractive for stereotactic radiotherapy (39). However, compared to VMAT-FF, the use of the higher mean dose rate of VMAT-FFF did not translate into a time advantage due to the limited speed of the gantry rotation (4.8 degrees per second on the Trilogy). There was no statistical difference for the passing rate of γ between the FF and FFF, which was in accord with these studies of left-sided breast cancer (40) and prostate cancer (19). Moreover, the VMAT plans demonstrated better consistency between the calculated dose distributions and the measured dose distributions than the IMRT technique, which might be because IMRT had more MUs and more complex modulation.

In this study, FFF plans tended to show a significant reduction in dose to healthy tissue compared with standard FF plans in view of the integral dose of non-tumor tissue, which may lead to reducing the risk of long-term radiation-induced complications (41). Reason for this may be that the main source of photon scatter in the treatment head is eliminated by removing the flattening filter, leading to a reduction in the out-of-field dose. Linac head leakage was reduced by 52% by using 6MV FFF beam for IMRT prostate treatment demonstrated by Kragl et al. (17). Simultaneously, many studies have shown that the unflattened mode delivers a much lower peripheral dose (17, 42). Besides, on account of the elimination of beam hardening components from the flattening filter, the spectrum of the unflattened 6 MV beam is usually softer (43). According to Vassiliev et al., 6 MV FFF beam has a depth dose distribution that is comparable to that of conventional 4–5 MV beam (44). As a result, the dose to the skin may be slightly higher (45). The results of this study indicate for WBRT with hippocampal avoidance that the reduced head scattering and residual electron contamination are predominant in patient dose reduction and beam softening does not cause excessive phantom scattering, at short-to-medium distances from the field edges.

This study still has several limitations. For example, the number of patients is small, and this study is based on dosimetry and radiobiology rather than clinical outcomes. Hence, the practical benefits of the FFF plans need to be confirmed by long-term follow-up and a larger number of cases before the FFF mode is widely employed for HA-WBRT, which is the subject of further study. Besides, due to the lack of measuring equipment in our institution, the integral dose was used to compare the out-of-field doses between FF and FFF beams in this paper. However, the peripheral doses are difficult to calculate correctly with the TPS, and the determination of peripheral doses by measurements of thermoluminescent dosimeters is more recommended (46, 47). In addition, several novel approaches have been proposed for HA-WBRT and have shown promising dosimetric advantages (48, 49). The combination of these methods and FFF techniques is expected to improve treatment plans, which also requires further investigation. Despite these limitations, our study will contribute to understanding the differences between the unflattened and flattened beams in whole-brain radiotherapy with hippocampal avoidance.

## 5 Conclusions

This study is the first to present evidence of the possible benefits of using FFF beams in HA-WBRT in terms of dosimetry and radiobiology, which is important to provide a new idea for improving the efficacy and neurocognitive side effects of HA-WBRT. VMAT with FFF beams achieved superior homogeneity and conformity of the target, better sparing of OARs, and lower NTCP of hippocampus with the similar treatment time compared to flat beams. The improvement resulting from the FFF technique in VMAT increased as the volume of the hippocampus decreased. IMRT-FFF provided comparable plan quality to IMRT-FF with the significantly reduced delivery time. Hence, FFF had a greater dosimetric effect on VMAT than IMRT. In addition, FFF beams showed a lower out-of-field dose, which may lead to reducing secondary cancer risk. FFF plans necessitated a significant increase in monitor units per fraction dose for both IMRT and VMAT, which was associated with the unflattened profile of FFF beams. The gamma scores of all plans were up to standard and no significant differences were detected between FF and FFF. Besides, VMAT showed considerable advantages over IMRT in terms of the plan quality, monitor units, treatment time and gamma indices. To sum up, VMAT-FFF offers the greatest dosimetric and radiobiological superiority, as well as the shortest treatment time compared to other techniques, so it may be considered the preferable therapeutic schedule for HA-WBRT.

## Data availability statement

The raw data supporting the conclusions of this article will be made available by the authors, without undue reservation.

## Ethics statement

The studies involving humans were approved by the ethics committee of Affiliated Hospital of Hebei University. The studies were conducted in accordance with the local legislation and institutional requirements. Written informed consent for participation was not required from the participants or the participants’ legal guardians/next of kin in accordance with the national legislation and institutional requirements. Written informed consent was obtained from the individual(s) for the publication of any potentially identifiable images or data included in this article.

## Author contributions

FL: Conceptualization, Funding acquisition, Project administration, Writing – original draft. YP: Investigation, Methodology, Writing – review & editing. QL: Validation, Writing – review & editing. QZ: Data curation, Writing – review & editing. HS: Data curation, Writing – review & editing. SQ: Writing – review & editing. RZ: Writing – review & editing.
